# Perinatal midwifery care demand in a tertiary hospital: A time-series analysis

**DOI:** 10.1016/j.ijnsa.2025.100299

**Published:** 2025-01-21

**Authors:** Luisa C. Eggenschwiler, Giusi Moffa, Valerie Smith, Michael Simon

**Affiliations:** a Department Public Health (DPH), Institute of Nursing Science (INS), University of Basel, Basel, Switzerland; b Chief Medical and Nursing Office, University Hospital Basel, Basel, Switzerland; c Department of Mathematics and Computer Science, University of Basel, Basel, Switzerland; d College of Health and Agricultural Sciences, School of Nursing, Midwifery, and Health Systems, University College Dublin, Belfield, Ireland

**Keywords:** Midwifery staffing, Nurse staffing, Workload, Workforce, Electronic health records, Routinely collected health data, Health services research

## Abstract

**Introduction:**

The chronic shortage of registered midwives and nurses is a serious global problem. However, current recommendations regarding midwifery staffing do not address operational staffing difficulties that arise from wide variations in care demand. The aim of this study was to describe shift-level care demand and available staffing resources in a tertiary hospital's maternity department.

**Methods:**

This single-centre retrospective longitudinal study investigated a four-year timeframe (2019–2022). All registered midwives and nurses working a three-shift pattern in the prenatal unit, labour ward, or postnatal unit were included. To determine care demand, we approached it in a novel way, accounting for both the number of women on each unit and each case's expected complexity. Any unmet care demand was calculated in relation to pre-specified nurse-to-patient ratios for each care area by subtracting demand hours from available staff hours per shift.

**Results:**

In total, 17,558 cases were included and 13,149 worked shifts analysed. The match of staffing resources with care demand was different for each analysed unit. In the prenatal and postnatal units, demand was generally met; however, the labour ward had a shortfall of at least one midwife on 32% of all shifts. Adjusted for care complexity, the deficiency prevalence rose to 55% of shifts for this ward.

**Conclusion:**

Alongside the inclusion of care complexity in assessing care demand, shift- and unit-level analyses showed that average staffing numbers obscure the actual volume of unmet care demand. Staffing in labour wards needs greater flexibility to cope with the clustering of births over short periods.

**What is already known about the topic**

• Maternity care demand varies over the course of years, weeks, and even shifts.
• Maternity care demand highly depends on case complexity.

Alt-text: Unlabelled box

**What this paper adds**

• The match between staffing resources and maternity care demand varies over time.
• Weighted maternity care demand based on case complexity revealed a higher mismatch over time.
• Understanding of care demand variation with regard to staffing resources provides an important perspective to improve allocation of limited resources.

Alt-text: Unlabelled box

## 1 Introduction

As international midwife and nurse staffing shortages grow (United Nations Population Fund et al., 2021), research is urgently needed to ensure safe maternity care (National Institute for Health and Care Excellence, 2015). Researchers have suggested direct links between higher levels of midwifery staffing and improved outcomes, including rates of exclusive breastfeeding and of births with an intact perineum, as well as higher maternal satisfaction (Turner et al., 2021). However, to understand what higher levels of midwifery staffing might look like, current staffing practices require further investigation (National Institute for Health and Care Excellence, 2015).

### 1.1 Care demand

To understand what constitutes adequate levels of staffing in the context of midwifery, one must first understand the care needs of pregnant and postpartum women and their newborns. As physiological birth is a process that follows its own schedule, numbers of women requiring maternity care can vary widely across years, weeks, and shifts (Deutsche Gesellschaft für Gynäkologie und Geburtshilfe e.V & Deutsche Gesellschaft für Hebammenwissenschaft e.V, 2020; Yelland et al., 2013). On maternity units, care demand can be measured as the sum of all care required by pregnant women, mothers and their newborns during their hospital stay (International Confederation of Midwives, 2019). Varying considerably between the pregnancy, labour, and postnatal periods, demand also reflects the complexity of each maternal trajectory.

Each case's complexity is affected by diverse factors, including conditions during pregnancy (e.g., preeclampsia), complications during labour (e.g., prolonged labour), and difficulties in the immediate postnatal period for the mother-newborn dyad (e.g., breastfeeding problems). At the unit level, both the number of women and their case complexity vary broadly (Allios et al., 2014).

### 1.2 Staffing

Staffing is the number of qualified care providers—normally registered midwives and registered nurses (RNs)—available during each time unit; e.g., one shift (Creswell et al., 2023). Based on long-term (e.g., yearly) care demand averages, each shift is allocated a specific number of registered midwives and RNs. In contrast to other countries (e.g., the United Kingdom (UK) and Australia), Swiss postnatal units are staffed mainly with RNs; considering the aforementioned shortages, registered midwives are distributed thinly (Royal College of Midwives, 2022; The Parliament of Victoria, 2022).

### 1.3 Matching staffing resources with care demand

If the planned staffing resources match the predicted demand, safety and quality of care are expected to be maintained. However, because of fixed roster planning, available staff cannot always respond adequately to fluctuations in maternity care demand. Current research on care demand and staffing is aggregated by place and time; e.g., at the hospital level (Clark et al., 2013; Turner et al., 2022) and based on annual birth numbers (Kpéa et al., 2015). With such broad aggregations, unit- and shift-level fluctuations and especially mismatches are underestimated, and the degree of unmet care demand remains unclear (National Institute for Health and Care Excellence, 2015; Turner et al., 2021).

#### 1.3.1 International staffing recommendations

To ensure safe staffing levels in the UK, the National Institute for Health and Care Excellence and the Royal College of Midwives have formulated recommendations for British maternity settings (National Institute for Health and Care Excellence, 2015; Royal College of Midwives, 2022). These follow the international gold standard of one-to-one care during established labour and birth (Deutsche Gesellschaft für Gynäkologie und Geburtshilfe e.V & Deutsche Gesellschaft für Hebammenwissenschaft e.V, 2020; National Institute for Health and Care Excellence, 2023; The Parliament of Victoria, 2022). The Royal College of Midwives also recommends implementing the Birthrate Plus® instrument to calculate the needed full-time equivalents of registered midwives to cover the target hospital's current average case mix and provide one-to-one care. In short, this instrument uses a scoring system to assign different weights to women's cases (care demand) and calculates the required midwife hours (Ball et al., 2013; Ball and Washbrook, 2010). While the National Institute for Health and Care Excellence guidelines provide structural level staffing recommendations in terms of full-time equivalents (National Institute for Health and Care Excellence, 2015), the Australian state of Victoria has introduced staff rostering regulations that specify shift- and unit-level midwife-to-woman ratios (The Parliament of Victoria, 2022).

In Switzerland, neither British- nor Australian-style staffing guidance is available. The current scarcity of national-level guidance reflects the limited understanding of complexity-based variation in care demand. As a result, Swiss maternity units’ staffing issues urgently need attention so that current and projected staffing shortages can be managed. Therefore, the overall aim of this time-series study was to explore and describe care demand and staffing resources in a tertiary maternity department. Its objectives were: (i) to describe complexity-weighted and unweighted care demand hours per pregnant woman, mother and newborn at the unit and shift level; (ii) to examine registered midwives’ and RNs’ unit- and shift-level staffing hours; and (iii) to describe unit- and shift-level matches between (unweighted and weighted) care demand and staffing.

## 2 Methods

### 2.1 Study design and setting

In Switzerland, forecasts indicate that midwifery and nursing care demand will soon outstrip workforce supply (Rüesch et al., 2014). Switzerland has an annual birth rate of around 10/1,000 inhabitants (Federal Statistical Office, 2024), all of which are attended by registered midwives. The vast majority (98%) of births take place in acute care hospitals (Federal Statistical Office, 2019), with registered midwives acting as lead health care professional for physiological births and working in close collaboration with obstetricians for non-physiological births. Mothers usually remain in postnatal units for three to four days. Post-discharge, they receive care at home for up to eight weeks from independent community registered midwives. This retrospective single-centre observational study was conducted in a tertiary hospital's maternity department with around 2,600 births per year. The study period regarding staffing was four years, from 01 January, 2019 to 31 December, 2022.

The maternity department operated three inpatient units: prenatal (Unit 1), labour (Unit 2), and postnatal (Unit 3). Units 1 and 2 were staffed entirely by registered midwives; Unit 3 was staffed mainly by RNs, with some registered midwives (9%). Their staffs rotated through day (07:00–16:00), late (14:30–23:00) and night (22:30–07:30) shifts; no other shift pattern was in operation. The three units were fully independent; i.e., staff did not work or rotate across units. There is a pool of RNs associated with Unit 3, which allows planning for additional staff who are familiar with the unit. There are no additional resources to address unusually high demand.

### 2.2 Sample

#### 2.2.1 Care demand

Pregnant women, mothers and newborns in the three studied units were included if they had at least one inpatient entry during the four-year study period. Because some mothers give birth to more than one child over four years, the number of inpatient admissions (cases), rather than the number of women (individual-level), was later included. All outpatient visits to Unit 2 during the target timeframe were also selected. Outpatient visits to Unit 2 occurred for emergency observation (e.g., for early contractions) and external cephalic versions.

#### 2.2.2 Staffing

**Fig. 1:**
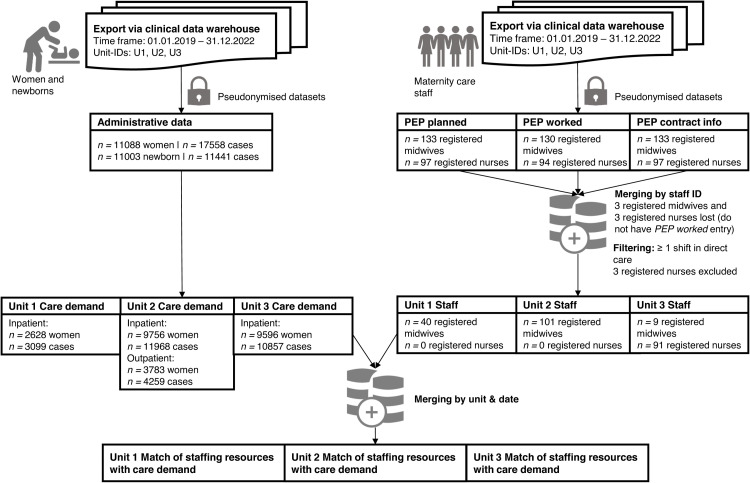
Data source linkage process and sample flow diagram. ID = identifier, PEP = PEP® software, which is used for staff rostering and reimbursement.

All registered midwives and RNs providing care on the three selected units during the study timeframe were included, as identified through the staffing roster with assigned shifts in direct care. Registered midwives and RNs with leadership positions were counted as supernumeraries during leadership duties and were included only when they were scheduled for direct care provision. Nurse assistants, students, and other medical staff were excluded. The linkage and process of sample selection are displayed in Fig. 1.

### 2.3 Variables, data sources and measurements

#### 2.3.1 Care demand

Based on care needs, care demand was determined by summing care hours per shift and unit. *Care demand in hours per shift* per case was calculated by the duration (in hours) of a woman's presence on a unit multiplied by her complexity factor (described below). Care demand information was extracted from administrative data (Federal Statistical Office, 2020). Variables such as main and secondary diagnoses (version ICD-10-GM), procedure codes, and diagnosis-related groups are routinely recorded for invoicing and statistical purposes (Federal Statistical Office, 2020). Details regarding variables and data sources are displayed in Supplementary Material Table S1.

#### 2.3.2 Complexity factor

To assess care demand in relation to care needs, we developed a complexity factor. Inspired by Birthrate Plus®, the complexity factor includes pregnancy, labour, and newborn complications (Ball et al., 2013). We assigned three prenatal categories: A1, a low-complexity prenatal complication (e.g., preterm contractions which were stable); A2, higher-complexity complications; and A3, perinatal deaths and abortions. A fourth category—for the prenatal unit—was inductions (I1), which were initiated in the prenatal unit. Each labouring women was designated one of five levels of labour complexity (L1–L5). L1 cases were least complex, with one-to-one care and a complication-free birth; L5 cases were the most complex, requiring more than one registered midwife throughout labour and birth. As these categories also factored in newborns’ care complexity, newborns were not counted as separate cases (Table 1). Similarly, the postnatal unit had two additional categories: one for readmissions (R1), another for gynaecological patients (G1). Each complexity factor was determined based on diagnoses, procedures, and diagnosis-related groups (see Supplementary Material Table S2 & Table S3 (Ball et al., 2013; National Institute for Health and Care Excellence, 2023)). To account for the complexity of each category, we assigned weights (Table 1) with categories L3 requiring 20%, L4 30% and L5 40% more care resources than categories L1 and L2 (Ball et al., 2013).

**Table 1:** Complexity factor categories.

Complexity factor	Description	Units	Weight
Category A1 (antenatal)	Pregnant women who need monitoring but are in a stable situation.	Unit 1 Unit 2 Unit 3	1 0.5 1
Category A2 (antenatal)	Pregnant women who need increased monitoring and can become unstable quickly; e.g., preeclampsia, vaginal bleeding.	Unit 1 Unit 2 Unit 3	1.4 1 1
Category A3 (antenatal)	Abortions, perinatal deaths before, during and after birth (if birth occurred in the prenatal unit).	Unit 1	2
Category L1 (labour)	Spontaneous vaginal birth without complications	Unit 2 Unit 3	1 1
Category L2 (labour)	Spontaneous vaginal birth with perineal tear or use of epidural anaesthetics	Unit 2 Unit 3	1 1
Category L3 (labour)	Spontaneous vaginal birth with more complications or instrumental birth without further complications	Unit 2 Unit 3	1.2 1.2
Category L4 (labour)	Instrumental births, planned caesarean sections and spontaneous vaginal births with postpartum emergencies; e.g., postpartum haemorrhage.	Unit 2 Unit 3	1.3 1.3
Category L5 (labour)	All perinatal deaths and abortions, emergency caesarean sections; all births requiring additional care due to complications.	Unit 2 Unit 3	1.4 1.4
Category I1 (inductions)	Pregnant women who need an induction and no room is available in the labour ward. They stay in the prenatal unit until labour is established.	Unit 1	2
Category R1 (readmissions)	Mothers who are readmitted to hospital due to complications after birth.	Unit 2 Unit 3	0.5 1
Category G1 (gynaecological)	Patients with gynaecological diagnoses who are transferred to the postnatal unit due to capacity utilisation.	Unit 1 Unit 3	1 1

Unit 1: prenatal unit; Unit 2: labour ward; Unit 3: postnatal unit

For Units 1 and 3, care demand changes throughout the day, decreasing during usual sleep periods. To incorporate the daily fluctuations, a target registered midwife/RN-to-mother-newborn dyad ratio was defined per unit and per shift (Supplementary Material Table S4), based on target ratios recommended by the (Australian) Victoria regulations noted above (The Parliament of Victoria, 2022). They recommend for prenatal units (here Unit 1) a ratio of 1:4 for day and late shifts and a ratio of 1:6 for the night shift. For postnatal units (here Unit 3), they recommend the same ratios: 1:4 in day and late shift, 1:6 in night shift.

#### 2.3.3 Staffing

Staffing hours were the product of the number of registered midwives combined with RNs, multiplied by the shift duration (in hours). Information was extracted from the PEP® software, which is used for staff rostering and reimbursement (POLYPOINT AG, 2024) and includes data on which care providers worked where (unit) and when (shift).

#### 2.3.4 Matching staffing resources with care demand

To determine the match of staffing resources to care demand, we subtracted the number of care demand hours from the number of staff hours per shift and unit. To incorporate the unit specific target ratios, we first divided the care demand hours by the ratio applied for the specific shift and unit, to then subtract those hours from staff hours. A difference of zero indicated matched care demand with resources (Supplementary Material Fig. S1).

### 2.4 Data access, linkage and cleaning methods

#### 2.4.1 Data access and cleaning methods

Data on women and newborns were extracted from the hospital data using unit identifiers for the three studied units over the study period (01 January 2019 – 31 December 2022). To determine which staff members worked at least one eligible shift, rosters were extracted by unit identifier for the target timeframe. The local nursing data scientist extracted all requested information from the clinical data warehouse, pseudonymised all identifiers, women, newborns, and staff members, thus deleting all identifying information (e.g., identifiers, names).

#### 2.4.2 Linkage

Pseudonymised data was provided as individual data sets (see Fig. 1). Three PEP® data sets (planned, worked, contract details) were merged by the staff identifier. For the final analysis, aggregated woman (care demand) and staffing data were merged by date and unit identifier. During the linkage process, for reasons provided in Fig. 1, some registered midwives and RNs were lost.

#### 2.4.3 Statistical methods

All statistical analyses were conducted using R (version 4.2.3) on Linux (Ubuntu 20.04) (R Core Team, 2024). We used the *tidyverse (dplyr, ggplot2, purrr), padr* and *lubridate* packages for data preparation, validation, and plotting (Grolemund and Wickham, 2011; Thoen, 2022; Wickham et al., 2019). To depict the variation in care demand, we calculated the number of births per day, a 14-day moving average, and assessed the coefficient of variation first for births per day, then for each type of birth per day (spontaneous vaginal birth, instrumental birth, planned caesarean section, and unplanned caesarean section). A high coefficient of variation is interpreted as high variability of data in relation to the mean. To form overviews of the units, we calculated the means, standard deviations (SDs), medians, and interquartile ranges (IQRs) of the numbers of care demand hours and staff hours.

Due to wide variations in care demand, each shift was assessed individually regarding staffing resources-demand match applying the assigned unit ratios (Supplementary Material Table S4). Calculations were performed using unweighted, then weighted care demand based on individual level complexity factors. Every eight hours of unmet care demand (-8 h) on a shift indicated one too few registered midwives/RNs working on that shift; every eight hours of surplus staffing resources (+8 h), indicated one unneeded registered midwife/RN.

## 3 Results

In this study, we included 11,088 women and 11,003 newborns, representing 17,558 woman cases and 11,441 newborn cases (Fig. 1). We also included 130 registered midwives, who worked 32,959 shifts, and 91 RNs, who worked 22,357 shifts.

### 3.1 Care demand—Births

**Fig. 2:**
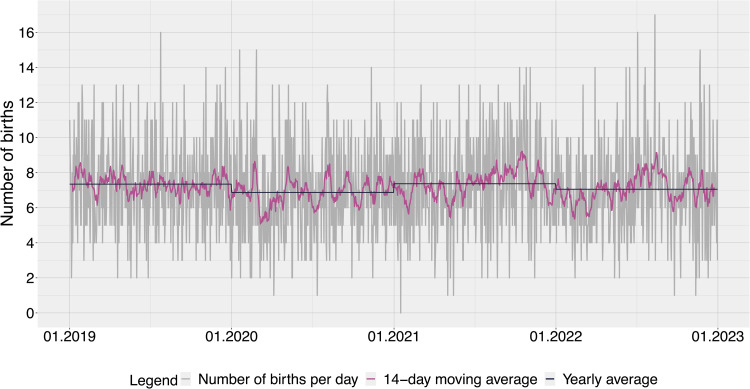
Variation of daily number of births. Number of births per day (grey), 14-day moving average (rose), yearly average (dark blue).

The daily number of births ranged from 0 to 17, with a median of seven births per day (IQR 5–9, mean 7.2, SD±2.6). In Fig. 2, the number of daily births, the 14-day moving average, and the yearly averages show variation in care demand (coefficient of variation 0.36) that accompanies the unpredictable clustering of births. During the study timeframe, the study hospital had an overall caesarean section rate of more than one-third, with 17.8% planned and 18.7% unplanned. All other types of birth similarly occurred in clusters (Supplementary Material Fig. S2). The coefficients of variation were lowest for spontaneous vaginal birth (0.52) and highest for instrumental vaginal birth (0.98), with planned and unplanned caesarean sections placed between the two (respectively 0.87 and 0.85).

### 3.2 Care demand—Case mix per shift

In Unit 1, eight women were typically present per shift (IQR 7–10). Most pregnant women were considered clinically stable (median 5; IQR 3–6, category A1). Usually, though, one (IQR 1–2) had a high risk of becoming unstable (category A2). A typical shift would involve one woman being induced (IQR 0–2). Fortunately, few women were in category A3 (abortion or perinatal death, median 0; IQR 0–1).

Unit 2 served a median of six women per shift (IQR 4–7). Most were admitted for birth (median 5; IQR 4–7, categories L1–L5). The median for unstable prenatal cases and postnatal readmissions was zero (IQR 0–0). A typical shift included two births (IQR 1–3). In addition to standard inpatient care, four one-hour pregnancy checks were conducted per day (IQR 2–5).

Unit 3 typically cared for 22 women per shift (IQR 19–25). Of these, 20 were commonly post-birth (IQR 17–23) and one a postnatal readmission (IQR 0–2). Table 2 presents additional care demand characteristics. Whereas Unit 1’s median length of stay was two days (IQR 1–4) and Unit 2’s 10.3 h (IQR 6–21), postpartum women typically stayed three days in Unit 3 (IQR 2.2–3.5).

**Table 2:** Care demand characteristics.

Demand sample characteristics	Unit 1 (prenatal unit)	Unit 2 (labour ward)	Unit 3 (postnatal unit)
**Number of people**			
Inpatient woman	2628	9756	9595
Inpatient newborn	0	10814	9708
Outpatient woman	0	3783	0
**Number of cases**			
Inpatient woman	3099	11968	10857
Inpatient newborn	0	10820	9956
Outpatient woman	0	4259	0
**LOS, median, (IQR)**	*n=* 3099	*n=*11886*	*n=* 10857
Inpatient case, woman	2.1 (1-4) *days*	10.3 (6-21) *hours*	2.9 (2.2-3.5) *days*
Outpatient case, woman	-	*N=4259* 1 (1-1) *visits*	-
**Type of birth, n (%)**	-	*n=*10457	-
Vaginal birth		5193 (49.7%)	
Instrumental vaginal birth		1438 (13.8%)	
Planned caesarean section		1866 (17.8%)	
Unplanned caesarean section		1960 (18.7%)	
**Complexity Factor, n (%)**	*n=*3099	*n=*11968	*n=*10857⁎⁎⁎
Category			
L1	-	351 (2.9%)	322 (3.0%)
L2	-	1578 (13.2%)	1473 (13.6%)
L3	-	3204 (26.8%)	3089 (28.5%)
L4	-	3074 (25.7%)	2985 (27.5%)
L5	-	2313 (19.3%)⁎⁎	2117 (19.5%)
A1	1508 (48.7%)	1187 (9.9%)	61 (0.6%)
A2	478 (15.4%)	225 (1.9%)	6 (0.1%)
A3	237 (7.6%)	-	-
I1	852 (27.5%)	-	-
R1	8 (0.3%)	36 (0.3%)	655 (6.0%)
G1	16 (0.5%)		149 (1.4%)

Note: IQR = interquartile range, LOS = length of stay, n = number; complexity factor categories explained in Table 1

⁎ In 82 cases, missing data precluded LOS calculations.

⁎⁎ Abortions and perinatal deaths cared for in the labour ward (Unit 2) are all categorised in L5. Therefore, while 10,520 cases are categorised as L1–L5, only10,457 births were subject to notification. The remaining births were <23 pregnancy weeks and would not be included in the national statistics of births.

⁎⁎⁎ Fewer mothers stayed in the postnatal unit than there were births because some either transferred (at their request) to the birth centre for postnatal care or went directly home.

### 3.3 Staffing

A typical Unit 1 shift roster placed two registered midwives on the day shift (IQR 2–3), two on the late shift (IQR 2–2) and one on the night shift (IQR 1–1). Unit 2 typically staffed six registered midwives on the day shift (IQR 6–7), six on the late shift (IQR 5–6) and five on the night shift (IQR 5–5). Unit 3 usually provided seven registered midwives/RNs on the day shift (IQR 7–8), four on the late shift (IQR 4–4) and four on the night shift (IQR 4–4).

### 3.4 Main results

For a broad overview, including median numbers of hours of care demand (unweighted and weighted) and of staffing, as well as matching staffing resources with care demand (unweighted and weighted), see Table 3.

**Table 3:** Care demand hours, staff hours, and matching hours (medians, IQRs).

Unit & shift	Care demand hours* Median (IQR)	Care demand hours, weighted Median (IQR)	Staff hours Median (IQR)	Matching resources with demand hours Median (IQR)	Matching resources with demand hours, weighted Median (IQR)
Unit 1					
Day shift *n=*1461 Target ratio 1:4	15.5 (12.9 – 18.4)	19.9 (15.9 – 23.4)	15.5 (15.0 – 22.5)	+2.8 (-0.6 – 6.6)	-1.4 (-5.4 – 2.9)
Late shift *n=*1461 Target ratio 1:4	14.3 (11.6 – 17.2)	18.4 (14.7 – 21.9)	14.0 (14.0 – 14.8)	+0.3 (-2.3 – 3.5)	-3.5 (-7.3 – 0.2)
Night shift *n=*1461Target r atio 1:6	10.2 (8.5 – 12.4)	13.1 (10.6 – 15.7)	7.5 (7.5 – 8.0)	-2.5 (-4.5 – -0.4)	-5.3 (-7.8 – -2.8)
Night shift *n=*1461 Target ratio 1:8⁎⁎	7.6 (6.4 – 9.3)	9.8 (8.0 – 11.8)	7.5 (7.5 – 8.0)	+0.1 (-1.4 – 1.7)	-2.0 (-3.9 – 0.0)
Unit 2⁎⁎⁎					
Day shift *n=*1461 Target ratio 1:1	44.2 (34.3 – 53.4)	52.8 (41.4 – 65.1)	39.5 (36.5 – 44.0)	-3.8 (-12.8 – 4.8)	-12.9 (-24.3 – -1.8)
Late shift *n=*1461 Target ratio 1:1	38.4 (29.6 – 47.4)	46.5 (35.1 – 58.3)	40.5 (35.3 – 41.5)	+0.8 (-8.0 – 9.6)	-7.3 (-18.3 – 3.3)
Night shift *n=*1461 Target ratio 1:1	36.5 (27.3 – 45.0)	43.9 (32.9 – 55.8)	32.5 (32.5 – 33.5)	-2.8 (-11.7 – 5.4)	-10.2 (-21.6 – -0.1)
Unit 3					
Day shift *n=*1461 Target ratio 1:4	42.0 (36.6 – 47.0)	51.5 (44.9 – 57.8)	53.5 (49.8 – 61.0)	+12.8 (7.4 – 18.7)	+3.3 (-3.0 – 10.3)
Late shift *n=*1461 Target ratio 1:4	36.1 (31.2 – 40.8)	44.0 (38.1 – 50.2)	29.0 (29.0 – 30.3)	- 6.5 (-10.8 – -1.9)	-14.4 (-19.8 – -8.8)
Late shift *n=*1461 Target ratio 1:6⁎⁎	24.0 (20.8 – 27.2)	29.4 (25.4 – 33.5)	29.0 (29.0 – 30.3)	+5.4 (2.5 – 8.5)	+0.1 (-3.4 – 3.7)
Night shift *n=*1461 Target ratio 1:6	27.8 (24.2 – 31.2)	34.1 (29.7 – 38.3)	30.0 (30.0 – 30.5)	+2.4 (-0.7 – 5.4)	-4.0 (-7.7 – 0.0)

Note: IQR = interquartile range, n = number;

⁎ Newborns are excluded from the calculation. They are represented by the complexity factor, which includes care for the newborn.

⁎⁎ Unit specific target ratios implemented during study timeframe

⁎⁎⁎ Outpatient visits are included in care demand hours.

#### 3.4.1 Unweighted match of staffing resources with care demand

The number of shifts with high mismatch was small for Unit 1; 0.4% of shifts (n=19) had >8 h of unmet demand, and 6.9% (n=303) had >8 h of excess staff. In Unit 2, however, 32.0% of shifts (n=1,402) lacked at least one registered midwife, while 21.6% (n=946) had surplus registered midwives. And in Unit 3, 13.6% of shifts (n=598) had >8 h of unmet demand, and 28.1% (n=1,233) had surplus staff.

#### 3.4.2 Weighted match of staffing resources with care demand

Overall, the number of weighted care demand hours per shift was higher than that of the unweighted. In Unit 1, 19.1% of shifts (n=838) had >8 h of unmet demand, and 3.0% (n=133) excess staff. In Unit 2, understaffing was severe; 55.2% of shifts (n=2,421) had too few registered midwives, while only 11.9% (n=520) had too many. And in Unit 3, 37.7% of shifts (n=1651) had unmet demand, compared to 11.6% (n=507) with surplus staff.

The only predictable demand related to planned caesarean sections. Those occurred during weekdays and never on weekends. This led to an interesting pattern in Unit 3. Due to a length of stay of three days post caesarean, the number of women accumulated in this unit, which had the highest numbers of women mostly on Thursdays and Fridays (Supplementary Material Fig. S3).

Fig. 3 depicts the match of staffing resources with care demand for all Unit 2 day shifts in terms of numbers of registered midwives needed (8 h=1 registered midwife)—first unweighted, then weighted. The matching figures with all shifts and for all units are provided in Supplementary Material Figs. S4–S13.

**Fig. 3:**
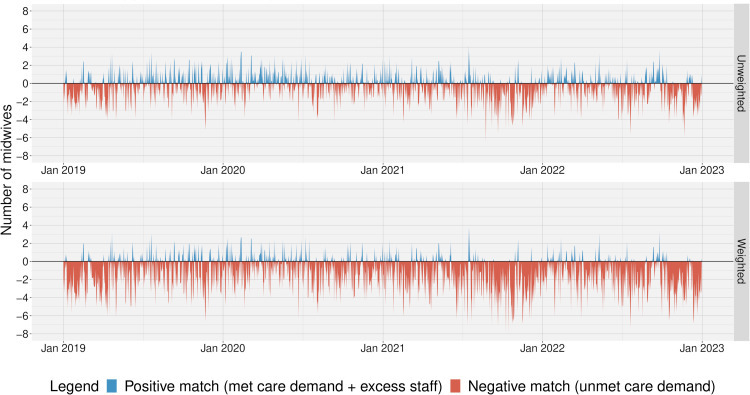
Match of staffing resources with care demand of all day shifts in Unit 2 (labour ward). Care demand is met at horizontal line = 0, number of registered midwives per day shift who are either not needed (blue) or demand that is not met (red) in Unit 2, once unweighted and once weighted with a complexity factor.

## 4 Discussion

In this study, we described shift- and unit-level care demand versus staffing in a Swiss tertiary hospital's maternity department. To address the extreme range of case-level care needs, we assigned each case a complexity factor. This allowed us to weight each case's needs proportionately to the hours of staff time required to meet them. In addition to inter-case differences in complexity, we found that much variation in staffing adequacy results from the clustering of births.

Jones and Hall (2022), in their study on labour ward care demand, also showed high variation in hourly demand during the day and for all weekdays. They further demonstrated that on Thursdays and Fridays, the demand was highest compared to other weekdays, which is similar to the demand in Unit 3 (postnatal unit) in our study. Because Jones and Hall (2022) conducted their study in the United States of America, where women generally stay only one day in the postnatal unit, this accumulation of women due to planned caesarean sections might be less severe than in Switzerland. Swiss researchers investigating patient turnover and patient-to-nurse ratios showed a similar pattern, although they drew together all maternity and gynaecological units, reducing the comparability to our unit-level differences (Musy et al., 2020). High variations in demand per shift (Creswell et al., 2023) and per month (Yelland et al., 2013) were also previously highlighted by researchers from Ireland and England, respectively, thus corroborating our results. Furthermore, we observed that, except for planned caesarean sections, all other types of birth and complexities occurred in clusters. Martin and colleagues (2018) observed similar patterns for planned caesarean sections. Their analysis of birth times indicated that spontaneous onset and birth occur more often during the night but that no specific patterns apply to instrumental births or unplanned caesarean sections (Macfarlane et al., 2019; Martin et al., 2018).

In assessing staffing, apart from shift-based differences, we found only relatively minor variations. Concerning the match of staffing resources with care demand, while both unweighted and weighted results varied greatly, weighting almost always increased the amount of unmet care demand. From a managerial perspective, the unweighted results indicated that Unit 1 (prenatal care) had sufficient staff, Unit 2 (labour ward) had a high share of mismatches but almost enough staff to ensure one-to-one care, and Unit 3 (postnatal care) had excess staff, with almost one-third of shifts having at least one surplus registered midwife/RN.

Newborns were not previously included in (unweighted) care demand calculations. Particularly for Unit 3, then, demand was not appropriately reflected (Matthews et al., 2024). However, because all case-level needs contribute to the complexity factor, weighted figures should reflect neonatal demand reasonably well. As a result, based on the match of staffing resources with weighted care demand, Unit 1 may have near-optimal staffing; it normally has a small share of excess capacity and demand goes unmet in only one fifth of shifts. For Unit 3, weighted figures indicated that in more than one third of shifts, staffing did not align well with the recommended ratios (Supplementary Material Table S4).

In contrast, fewer than half of Unit 2’s shifts were staffed adequately; i.e., for the majority, one-to-one care was not available for every woman in labour, and only one tenth of shifts indicated surplus capacity. Jones and Hall (2022) showed high variation in the match of staffing resources with care demand throughout the day and month, which was very similar to the unweighted Unit 2 (labour ward) results. When Siddiqui and colleagues (2014) applied complexity weighting to their data, they found a registered midwife shortage across shifts and days similar to that shown in our weighted Unit 2 results.

The results reported here indicate that applying a complexity factor to care demand data influences measures of how adequately that demand is met. It likely also explains differences between capacity utilisation measures of staffing adequacy, such as bed occupancy rate and frontline experience of it (García et al., 2022).

At operational levels in staff rostering, accurate assessment of unit- and shift-level matching of staffing resources with care demand can identify recurring and therefore systematic mismatches. In our example, obstetricians conducted all planned caesarean sections in the mornings from Monday to Friday. This regularly increased demand in Unit 3 on Thursdays and Fridays. To reduce this effect, either the planning of caesarean sections could be revisited and adapted or the late-week numbers of registered midwives/RNs could be adjusted to meet the higher demand and reduced on days when the unit is normally less busy; e.g., Monday. Jones and Hall (2022) implemented this strategy by adding rosters.

Furthermore, at the operational level, our Unit 2 (labour ward) staffing roster could be adjusted based on the numbers and due dates of women expecting to give birth there during a given period. In most Swiss hospitals, administrators receive this information six to eight weeks in advance. The additional responsiveness would demand greater flexibility but would reduce overloading. To further increase the match between staffing resources and care demand, the UK could serve as a role model in flexibly staffing labour wards to increase the likelihood of one-to-one care (APPG Baby Loss and Maternity, 2022). There are several challenges implementing this flexible staffing in Switzerland. On-call shifts are poorly paid and therefore often not accepted by the workforce to be implemented regularly (Arbeitsgruppe “Faire Pikett- und Überzeitentschädigung”, 2020). There are not enough registered midwives within a hospital for flexible staffing, as only few registered midwives work on the postanal unit; in our case, only 9%. RNs working on the postnatal unit receive further education in breastfeeding and caring for mothers and newborns to maintain quality of care. The main problem of this approach is that RNs cannot support the labour ward and be used as a buffer if demand exceeds capacity. Nonetheless, we believe that the study results are representative of large parts of the Swiss health care system. However, country-specific differences in the health care system need to be considered when interpreting this study's results.

A perspective that is not addressed in our analysis is deviations from the staffing roster. While the staffing roster is a strategic question and determines what could be achieved given the planned resources, the actual staffing, including sickness absences, overtime, and demand variation, points to operational staffing questions. In our analysis, the question remains open whether the mismatch arose because staffing did not follow the planned roster or whether planning was generally not suitable.

### 4.1 Limitations

To usefully interpret the results presented here, one must consider that the measures of complexity used were based on diagnostic lists compiled at discharge. At the structural level, they are useful to understand case mix and necessary numbers of full-time equivalents to provide one-to-one care but cannot not be used for operational planning. Nevertheless, we have provided a broad indication of factors influencing effective staffing. To apply complexity factors at an operational level, the complexity would have to be assessed prospectively and documented regularly; e.g., every six hours (Siddiqui et al., 2014).

Based on this information, two steps can be taken. One is to retrospectively analyse and search for regular care demand/staffing mismatches (e.g., repeated understaffing on Thursdays) and adjust the rostering accordingly. The second is to introduce an escalation algorithm (based on current mismatch levels) for short-notice staffing level adaptation (Government of Western Australia, 2020; Siddiqui et al., 2014).

The applied complexity factor resulted in weights applied in the calculations. These weights were inspired by Birthrate Plus® (Ball et al., 2013) and further staffing related studies (Jones and Hall, 2022; Siddiqui et al., 2014). We couldn't identify any literature recommending weights, either for the prenatal or for the postnatal unit. We collaborated with the clinical staff, who reviewed the suggested weights and confirmed those. To provide comparable weights for all three units, we took the target ratios into account while assigning the weights. For example, the category A3 in Unit 1 had a weight of two. Combined with the target ratio of 1:4 in the dayshift, the category A3 got four hours from a registered midwife during the day, compared to two hours for category A1. The advantages of weights are the representation of case complexity. Our weights were not validated, while Griffiths et al. (2023) also showed that Birthrate Plus® weights are not properly validated as well. Therefore, a validation of the weights is deemed necessary to draw stronger conclusions. In this study, the true workload might lie somewhere in-between the unweighted and weighted matches, which still showed substantial understaffing for Unit 2.

This analysis included all direct-care registered midwives and RNs in the study units. All supernumerary registered midwives and RNs, as well as nurse assistants and students, were excluded. Also, physicians were excluded from this analysis. To adhere to international guideline that every woman should receive one-to-one care during labour and birth, no additional staff should be included in calculations regarding registered midwife and RN staffing (Deutsche Gesellschaft für Perinatale Medizin, 2021; National Institute for Health and Care Excellence, 2015). Nevertheless, these additional staff provide critical support for direct care; e.g., leadership, teaching, and ancillary tasks.

Further aspects excluded from the analysis included registered midwife/RN experience and overtime. Experience in the field of maternity care, in the hospital, and on the unit might impact the quality of care provided (Matthews et al., 2024). Overtime hours were included without separate analysis. Especially in the labour ward spanning the late-shift-to-night-shift changeover, certain care demands were addressed through overtime.

Also, primarily when there was less work than usual, the study hospital used on-call staff. The present time of on-call shifts was included in the analysis; however, their waiting time at home was not. In general, but especially near the end of a shift, unmet care demand had to be quite serious before on-call staff were called in. Therefore, while a call-in would add one registered midwife to the staff, that person's presence would coincide—misleadingly—with increases in unmet demand, although there would have been a registered midwife available.

Finally, during the included timeframe, the hospital was impacted by the COVID-19 pandemic. There were two lockdowns, one in spring 2020 and another one in winter 2021. During the time of the pandemic, partners were allowed to be present during birth and during a short time of the day in the postnatal unit. Except for those measures, care proceeded as normal across the maternity department. For these reasons, we did not explore any COVID-19 pandemic-specific impacts on staffing.

## 5 Conclusion

As this study shows, routine patient data are suitable for detailed and meaningful shift- and unit-level data exploration. Our analyses of care demand and especially of data weighting provide detailed accounts of where and when staff might be needed, rather than simply assessing annual birth numbers. Here, a validation study could help to increase trust in the applied weights. Still, considering the unpredictability of care demand, the noted limitations regarding staff flexibility, and the crucial importance of maternity units’ work, perinatal workforce planning remains extremely challenging. Addressing these constantly-fluctuating care needs will require innovative approaches to roster planning. For example, in spite of birth clustering, shared pregnancy assessment data and identification information some weeks in advance (e.g., by women's obstetricians) might improve prediction of staffing needs. Information regarding complexity-increasing factors (e.g., twin pregnancies or suspected complications) should also be considered in advance. Furthermore, new shifts could be created to address demand peaks throughout the day or week. Further research should also look into the effects of these severely understaffed shifts on maternal and neonatal outcomes.

## Ethical approval and consent to participate

In a clarification of responsibility, the relevant ethics committee declared this project outside the scope of the Human Research Act (Req -2022-00208); therefore ethical approval was waived. And as the study site has implemented a general consent agreement allowing researchers to use routine hospital data, no extra consent was necessary.

## Availability of data and material

The datasets used and analysed during the current study are available from the corresponding author upon reasonable request. Meta-data and R Code used are available on Zenodo (10.5281/zenodo.10808186).

## Funding statement

This work was supported by the Swiss National Science Foundation [proposal number P000PS_214709].

## CRediT authorship contribution statement

**Luisa C. Eggenschwiler:** Writing – review & editing, Writing – original draft, Visualization, Project administration, Methodology, Funding acquisition, Formal analysis, Data curation, Conceptualization. **Giusi Moffa:** Writing – review & editing, Validation, Supervision, Methodology, Investigation, Conceptualization. **Valerie Smith:** Writing – review & editing, Validation, Supervision, Conceptualization. **Michael Simon:** Writing – review & editing, Validation, Supervision, Methodology, Funding acquisition, Conceptualization.

## Declaration of competing interest

The authors declare the following financial interests/personal relationships which may be considered as potential competing interests: Luisa Eggenschwiler reports financial support was provided by Swiss National Science Foundation. If there are other authors, they declare that they have no known competing financial interests or personal relationships that could have appeared to influence the work reported in this paper.

## Appendix Supplementary materials

Image, application 1
