# Supplementary material for: Perinatal midwifery care demand in a tertiary hospital: A time-series analysis
Source: Int J Nurs Stud Adv. 2025 Jan 21;8:100299. doi: 10.1016/j.ijnsa.2025.100299 (PMC11802361; doi:10.1016/j.ijnsa.2025.100299)
Supplement: Supplementary file 1 [file mmc1.pdf]

Supplementary Material

Table S1. Variables and data sources

Variable	Data source
Care demand	
Admission date & time	administrative data
Discharge date & time	administrative data
Transfer date & time	administrative data
Birth date	administrative data
Main & secondary diagnoses (ICD-10-GM)	administrative data
Main & secondary procedures (CHOP)	administrative data
Diagnosis-related groups (SwissDRG)	administrative data
Type of visit (inpatient, outpatient)	administrative data
Patient-ID	administrative data
Case-ID	administrative data
Unit-ID	administrative data
Staffing resources	
Shift start date & time	PEP® schedule<br>PEP® worked
Shift end date & time	PEP® schedule<br>PEP® worked
Shift type (day, late, night)	PEP® schedule<br>PEP® worked
Profession (registered midwife, registered nurse)	PEP® contract info
Staff-ID	PEP® schedule<br>PEP® worked<br>PEP® contract info
Unit-ID	PEP® schedule<br>PEP® worked

CHOP = Swiss procedures classification, ICD = International Classification of Diseases, ID = identifier, PEP® = staff scheduling software

Table S2. Complexity factor based on diagnosis codes (ICD-10-GM 2022) and procedure codes (CHOP 2022)

**Variable**	**ICD Code 2022**<br>**CHOP Code 2022**	**ICD Description**<br>**CHOP Description**	**Score**
Gestational age	O09.7	> 41+0	1
	O09.6	36+1 – 41+0	1
	O09.5	33+1 – 36+0	2
	O09.4	25+1 – 33+0	3
	O09.3	19+1 – 25+0	3
	O09.2	13+1 – 19+0	3
	O09.1	5+0 – 13+0	3
	O09.0	< 5+0	3
Labour duration	-	-	1
	O63.0	Prolonged first stage	2
	O63.2	Prolonged birth of second twin / triplet	2
	O63.9	Prolonged birth not specified	2
Labour interventions	-	-	-
	CHOP93.92.15	Personal controlled analgesia with remifentanil	3
	CHOP03.91.11	Spinal / epidural analgesia	3
	CHOP03.91.21		3
	CHOP03.91.31		3
	O68.0 / O68.2	Complications due to fetal heart rate anomaly	3
Multiple pregnancy	-	-	-
	O30.0	Twins	2
	O30.1	Triplets	5
	O30.2	Quadruplets	5
	O30.8	Multiples	5
	O30.9	Multiples, not specified	5
Medical condition	All codes from Table S3	If one of the codes is present, then the score is given.	5
Mode of birth	O80	Vaginal birth	1
	CHOP73.59	Manually supported vaginal birth	1
	O81	Instrumental birth	2
	CHOP72f	Instrumental birth	2
	O32.1 + 73.59	Breech position	2
	O82	Elective caesarean section	3
	CHOP 74._X.10	Caesarean section primary	3
	CHOP 74._X.00	Caesarean section not specified	5
	CHOP 74._X.20	Caesarean section secondary	5
	CHOP 74._X.99	Caesarean section other	5
	CHOP 74.3	Extraction of fetus (uterine rupture)	5
Obstetric injury	-	-	1
	CHOP74f + O82	Sectio Caesarea	1
	O70.0	Perineal tear 1st degree	2
	O70.1	Perineal tear 2nd degree	2
	O71.8	Other tear, not specified	2

Variable	ICD Code 2022<br>CHOP Code 2022	ICD Description<br>CHOP Description	Score
	O70.2	Perineal tear 3rd degree	3
	O70.3	Perineal tear 4th degree	3
	O70.9	Perineal tear, not specified	3
	CHOP73.6	Episiotomy	3
	CHOP72.X1	Instrumental birth + episiotomy	3
Fetal asphyxia	-	-	1
	P21.9	Asphyxia not specified	2
	P21.1	Mild to moderate asphyxia	2
	P21.0	Severe asphyxia	3
Birth weight	Z38f	Healthy newborn	1
	P07.12	Birth weight 1500-2499	2
	P07.11	Birth weight 1250-1499	3
	P07.10	Birth weight 1000-1249	3
	P07.02	Birth weight 750-999	3
	P07.01	Birth weight 500-749	3
	P07.00	Birth weight <500	3
Congenital anomaly	Q00-Q99	Congenital anomalies	3
Perinatal death	Z37.1	Stillborn singleton	5
	Z37.3	Twins, one stillborn	5
	Z37.4	Twins, both stillborn	5
	Z37.6	Multiples, some stillborn	5
	Z37.7	Multiples, all stillborn	5
	DRG P60A / P60B	Died during hospital stay	5
Postnatal interventions	CHOP 99.04	Blood transfusion	5
	CHOP75.4	Manual placenta extraction	
	CHOP75.5	Suture of uterus rupture	
	CHOP75.6	Suture of other tears [excl. 75.69]	
	CHOP75.7-75.9	Further procedures	
	O72f	Postnatal haemorrhage needing therapy	
	O73f	Retained placenta	
	O71.0	Rupture of uterus before labour	
	O71.1	Rupture of uterus during labour	
	O71.2	Postpartum inversion of uterus	
	O71.3	Obstetric laceration of cervix	
Intensive care needs	CHOP99.B8.1	Intermediate care	5
	CHOP99.B7.1	Intensive unit care	5
Sum score		Score 6 = Category L1	
		Score 7 – 9 = Category L2	
		Score 10 – 13 = Category L3	
		Score 14 – 18 = Category L4	
		Score >= 19 = Category L5	

Table S3. Complexity factor – variable medical condition

**Disease area**	**Medical condition**	**ICD Code**	**ICD Description**
Cardiovascular	Confirmed cardiac disease	O99.4	Diseases of the circulatory system complicating pregnancy, childbirth and the puerperium
	Hypertensive disorders	O10<br>O11<br>O16	Pre-existing hypertension<br>Pre-existing hypertension with pre-eclampsia<br>Unspecified maternal hypertension
Respiratory	Asthma requiring an increase in treatment or hospital treatment & Cystic fibrosis	O99.5	Diseases of the respiratory system complicating pregnancy, childbirth and the puerperium
Haematological	Haemoglobinopathies – sickle-cell disease, beta-thalassaemia major	D55<br>D56<br>D57	Anaemia due to enzyme disorders<br>Thalassaemia<br>Sickle-cell disorders
	Immune thrombocytopenia purpura or other platelet disorder or platelet count below 100×109/litre &<br>Von Willebrand's disease	O99.1	Other diseases of the blood and blood-forming organs and certain disorders involving the immune mechanism complicating pregnancy, childbirth and the puerperium
Endocrine	Hyperthyroidism	O99.2	Endocrine, nutritional and metabolic diseases complicating pregnancy, childbirth and the puerperium
	Diabetes	O24.0<br>O24.1<br>O24.2<br>O24.3	Pre-existing type 1 diabetes mellitus<br>Pre-existing type 2 diabetes mellitus<br>Pre-existing malnutrition-related Diabetes mellitus<br>Pre-existing diabetes mellitus, unspecified
Infective	Risk factors associated with group B streptococcus	B95.1	Streptococcus, group B, as the cause of diseases classified to other chapters
	Hepatitis B/C with abnormal liver function tests	O98.4	Viral hepatitis complicating pregnancy, childbirth and the puerperium
	Carrier of/infected with HIV	O98.7	Human immunodeficiency virus [HIV] disease complicating pregnancy, childbirth and the puerperium
	Toxoplasmosis – women receiving treatment	B58	Toxoplasmosis
	Current active infection of chicken pox/rubella/genital herpes in the woman or baby	O26.4	Herpes gestationis

Disease area	Medical condition	ICD Code	ICD Description
	Tuberculosis under treatment	O98.0	Tuberculosis complicating pregnancy, childbirth and the puerperium
Immune	Systemic lupus erythematosus & Scleroderma	O99.8	Other specified diseases and conditions complicating pregnancy, childbirth and the puerperium
Renal	Abnormal renal function	O99.8	Same as above
	Renal disease requiring supervision by a renal specialist	O26.81	Renal disease related to pregnancy
Neurological	Epilepsy & Myasthenia gravis	O99.3	Mental disorders and diseases of the nervous system complicating pregnancy, childbirth and the puerperium
Gastrointestinal	Liver disease associated with current abnormal liver function tests	O99.6<br><br>O26.60<br>O26.68	Diseases of the digestive system complicating pregnancy, childbirth and the puerperium<br>Liver disorders in pregnancy, childbirth and the puerperium
Psychiatric	Psychiatric disorder requiring current inpatient care	O99.3	
Current pregnancy	Placenta praevia	O44.10<br>O44.11	Low implantation of placenta with haemorrhage<br>Placenta praevia with haemorrhage
	Pre-eclampsia or pregnancy-induced hypertension	O13<br>O14<br>O15	Gestational hypertension<br>Pre-eclampsia<br>Eclampsia
	Placental abruption	O45	Premature separation of placenta<br>[all from O45.0 – O45.9]
	Substance misuse	F11-F19	Opioids, cannabinoids, sedativas, cocaine, hallucinogens, tobacco, volatile solvents, multiple drug use
	Alcohol dependency requiring assessment or treatment	F10	Mental and behavioural disorders due to use of alcohol<br>[all from F10.0-F10.9]
	Onset of gestational diabetes	O24.4<br>O24.9	Diabetes mellitus arising in pregnancy<br>DM in pregnancy, unspecified
	Recurrent antepartum haemorrhage	O46	Antepartum haemorrhage, not elsewhere classified<br>[all from O46.0 – O46.9]

Table S4. Midwife/nurse to patient ratios per unit and shift

Unit	Shift	Midwife/nurse to mother-newborn dyad ratio*	Category	Weight
Unit 1	Day shift<br>Late shift<br>Night shift	1:4<br>1:4<br>1:6	Category A1	1
			Category A2	1.4
			Category A3	2
			Category I1	2
			Category R1	1
			Category G1	1
Unit 2	Day shift<br>Late shift<br>Night shift	1:1<br>1:1<br>1:1	Category L1	1
			Category L2	1
			Category L3	1.2
			Category L4	1.3
			Category L5	1.4
			Category A1	0.5
			Category A2	1
			Category R1	0.5
Unit 3	Day shift<br>Late shift<br>Night shift	1:4<br>1:4<br>1:6	Category L1	1
			Category L2	1
			Category L3	1.2
			Category L4	1.3
			Category L5	1.4
			Category A1	1
			Category A2	1
			Category R1	1
			Category G1	1

*Following the Safe Patient Care Act 2015 from the State of Victoria, Australia: Safe Patient Care (Nurse to Patient and Midwife to Patient Ratios) Act 2015

For example, a woman in category L5 has a weight of 1.4, indicating a care need of 1.4 registered midwives during her stay in the labour ward. If, for example, the woman stayed for five hours in the labour ward, she needed seven hours of registered midwife care (5 h * 1.4). She may have needed care from one registered midwife for four hours (4 h registered midwife care) and from three registered midwives for one hour (3*1 h registered midwife care) because of an emergency caesarean section, equating thus to 4 h+3 h = 7 h registered midwife care.

Figure S1

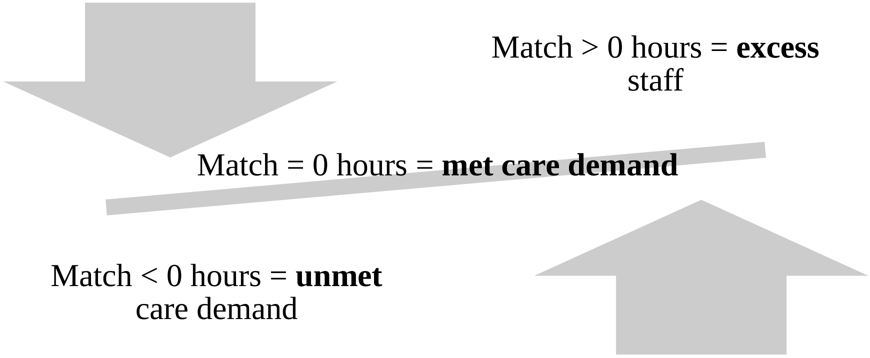

Figure S1 Matching staffing resources with care demand.

Figure S2

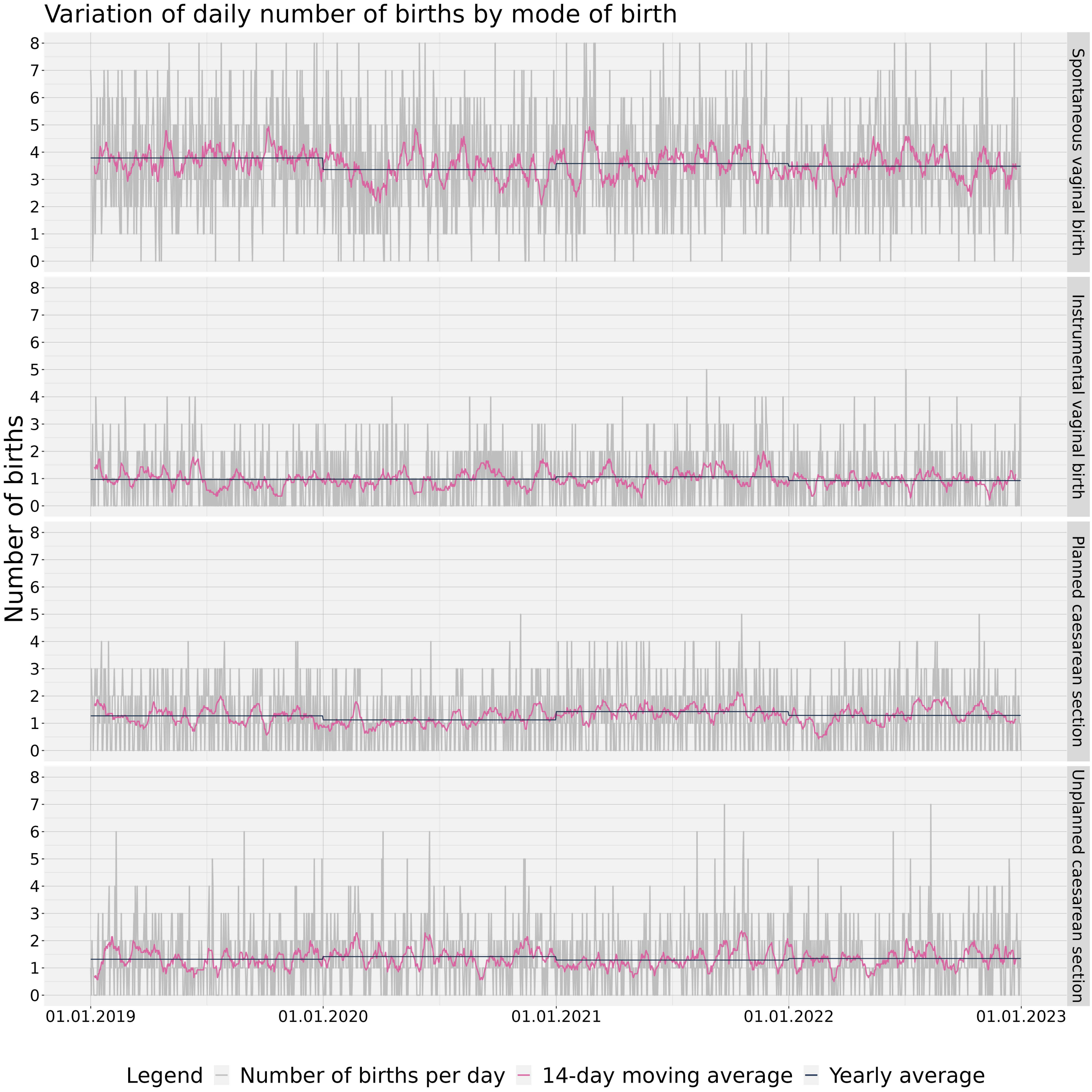

Figure S2 Variation of daily number of births by mode of birth. Number of births per day (grey), 14-day moving average (rose), yearly average (dark blue).

Figure S3

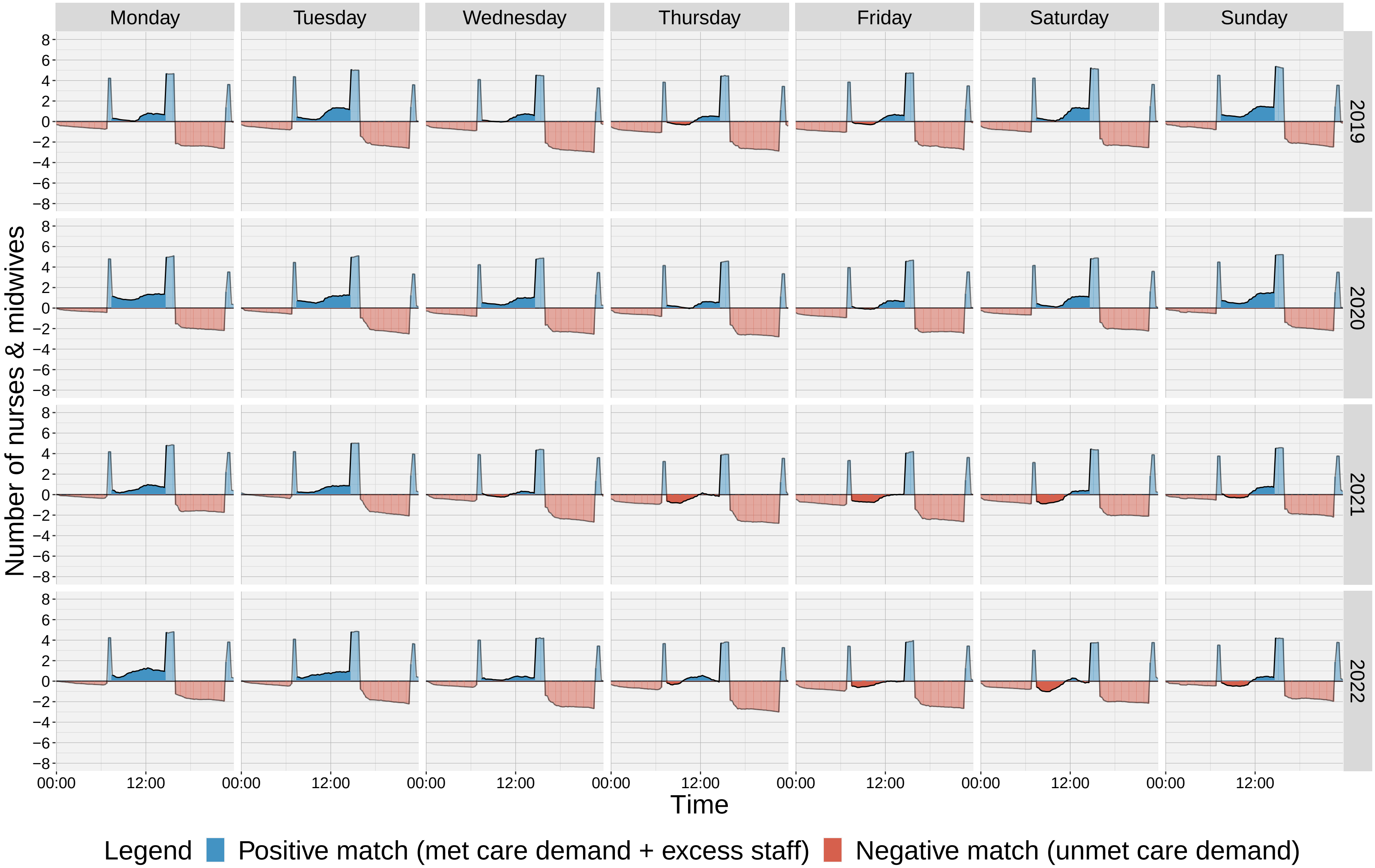

Figure S3. **Match of staffing resources with weighted care demand over days of week in Unit 3 (postnatal unit).** Care demand is met at horizontal line = 0, number of registered midwives / nurses per shift who are either not needed (blue), or demand that is not met (red) in Unit 3, weighted. The mean over days of the week and per year is displayed over time of the day starting at midnight and ending at midnight. The peaks represent handovers between the three shifts.

Figure S4

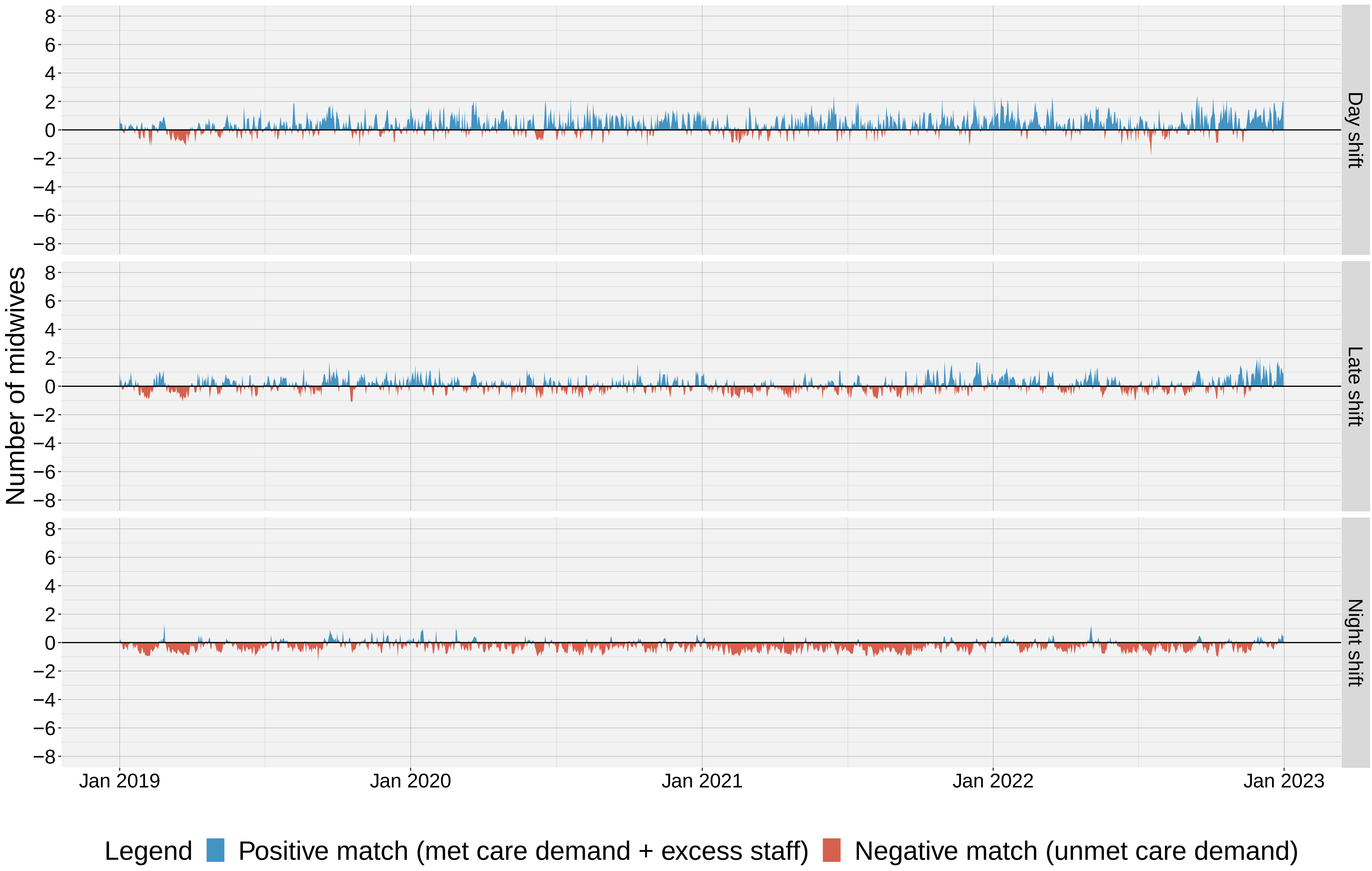

Figure S4 Match of staffing resources with unweighted care demand in Unit 1 (prenatal unit). Target ratios day shift 1:4, late shift 1:4, night shift 1:6, care demand is met at horizontal line = 0, number of registered midwives per shift who are either not needed (blue), or demand that is not met (red) in Unit 1, unweighted.

Figure S5

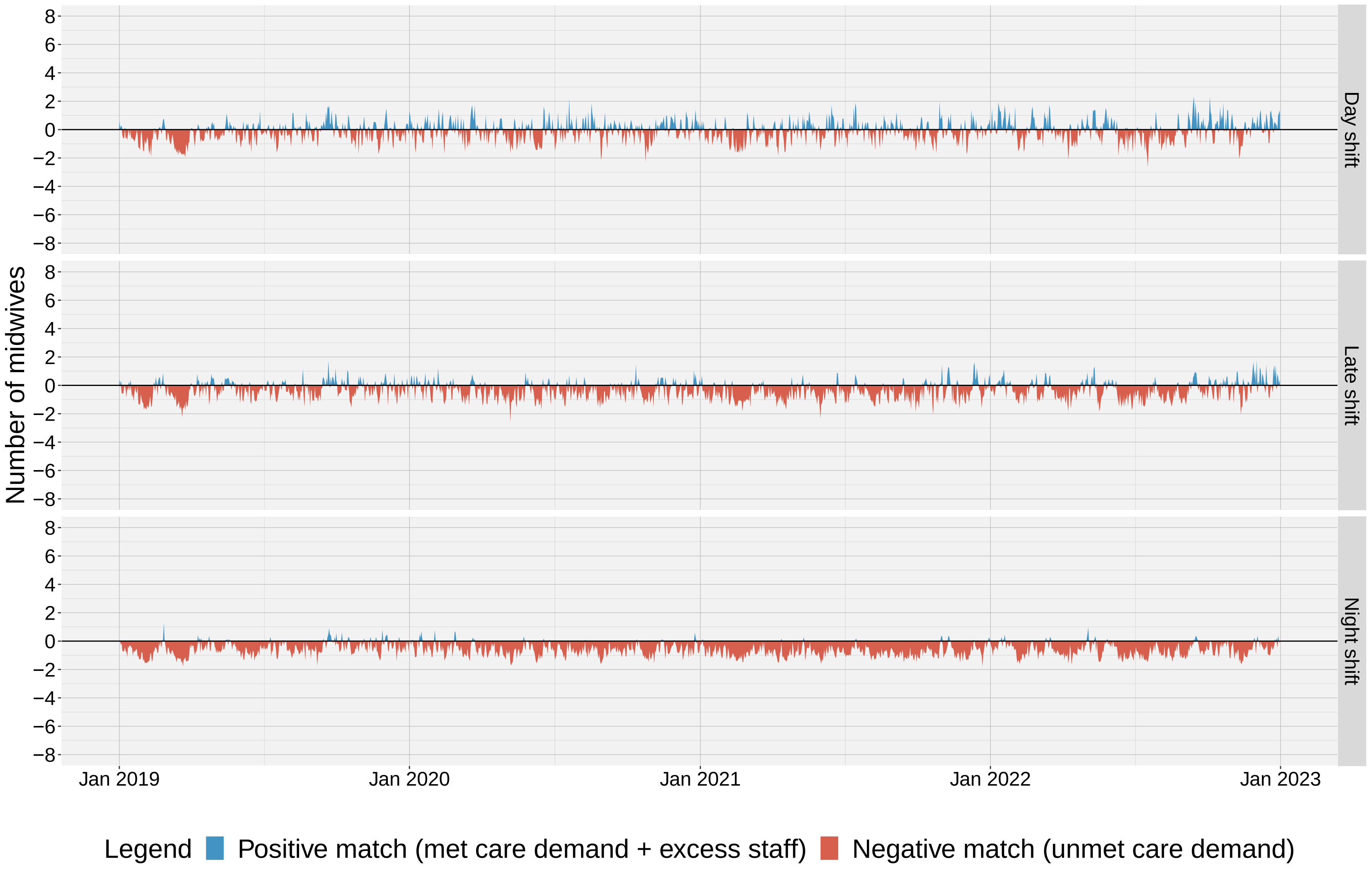

Figure S5 Match of staffing resources with weighted care demand in Unit 1 (prenatal unit). Target ratios day shift 1:4, late shift 1:4, night shift 1:6, care demand is met at horizontal line = 0, number of registered midwives per shift who are either not needed (blue), or demand that is not met (red) in Unit 1, weighted with complexity factor.

Figure S6

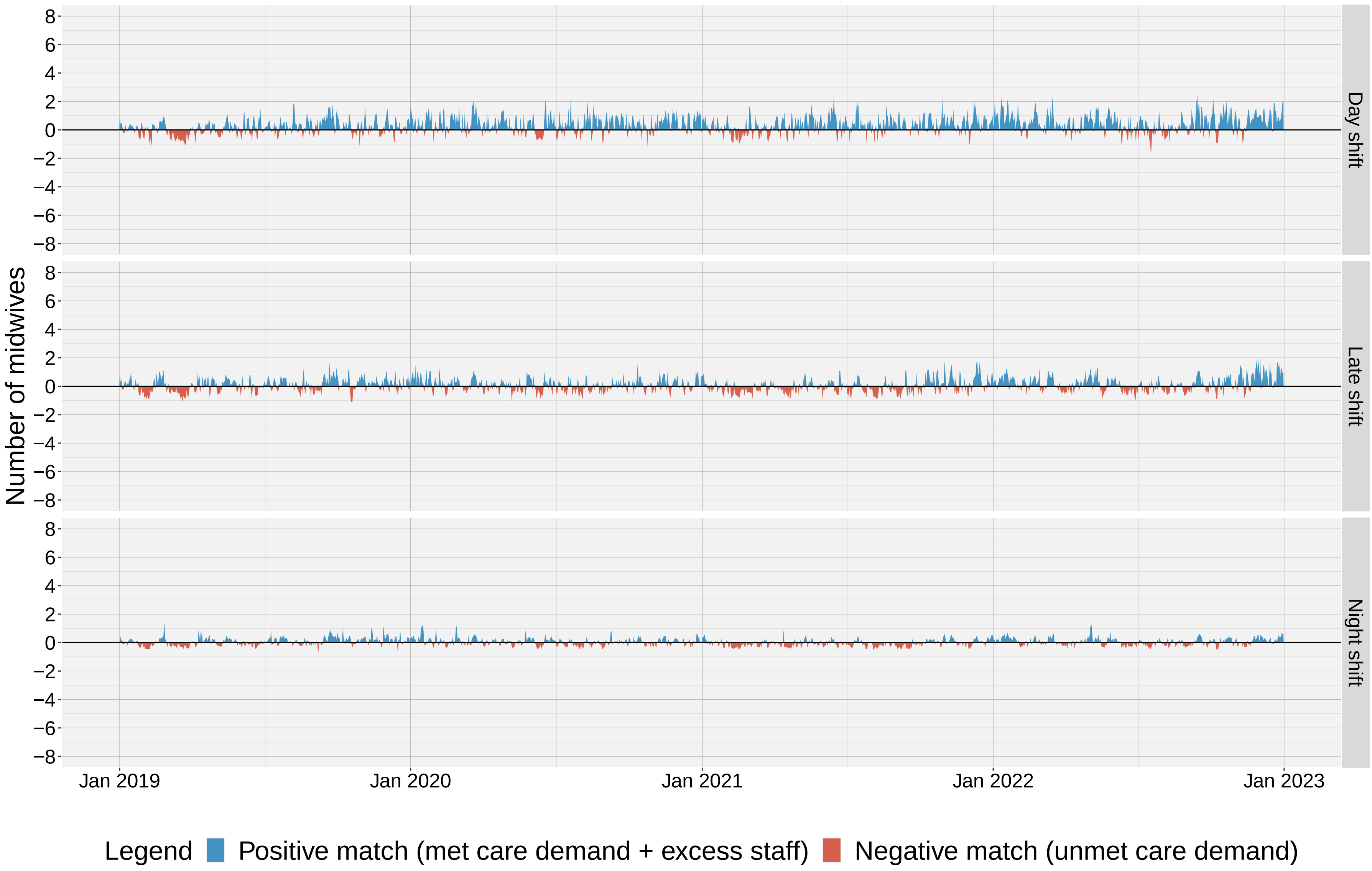

Figure S6 Match of staffing resources with unweighted care demand in Unit 1 (prenatal unit, unit-specific ratios). Target ratios day shift 1:4, late shift 1:4, night shift 1:8, care demand is met at horizontal line = 0, number of registered midwives per shift who are either not needed (blue), or demand that is not met (red) in Unit 1, unweighted.

Figure S7

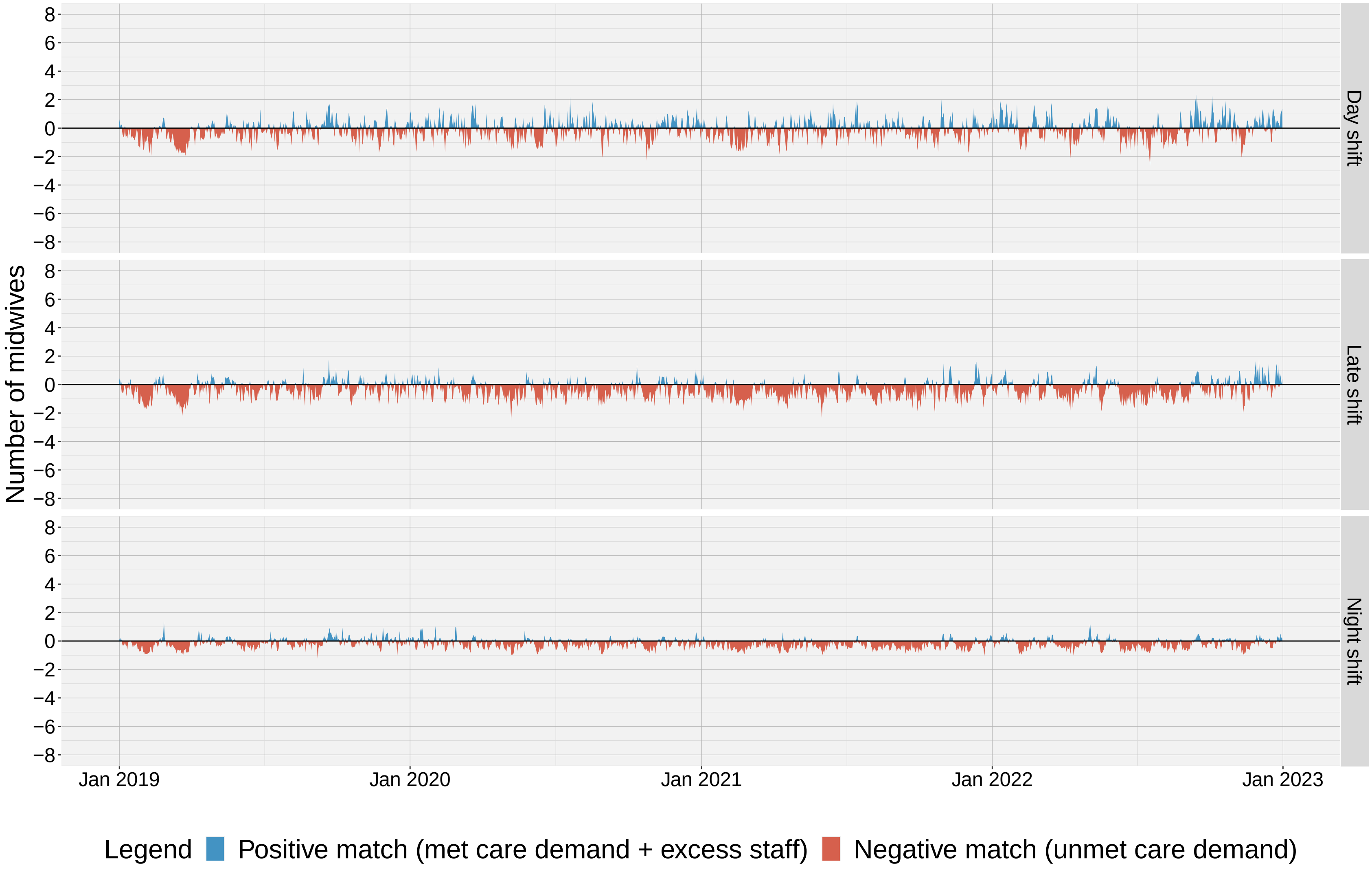

Figure S7 Match of staffing resources with weighted care demand in Unit 1 (prenatal unit, unit-specific ratios). Target ratios day shift 1:4, late shift 1:4, night shift 1:8, care demand is met at horizontal line = 0, number of registered midwives per shift who are either not needed (blue), or demand that is not met (red) in Unit 1, weighted with complexity factor.

Figure S8

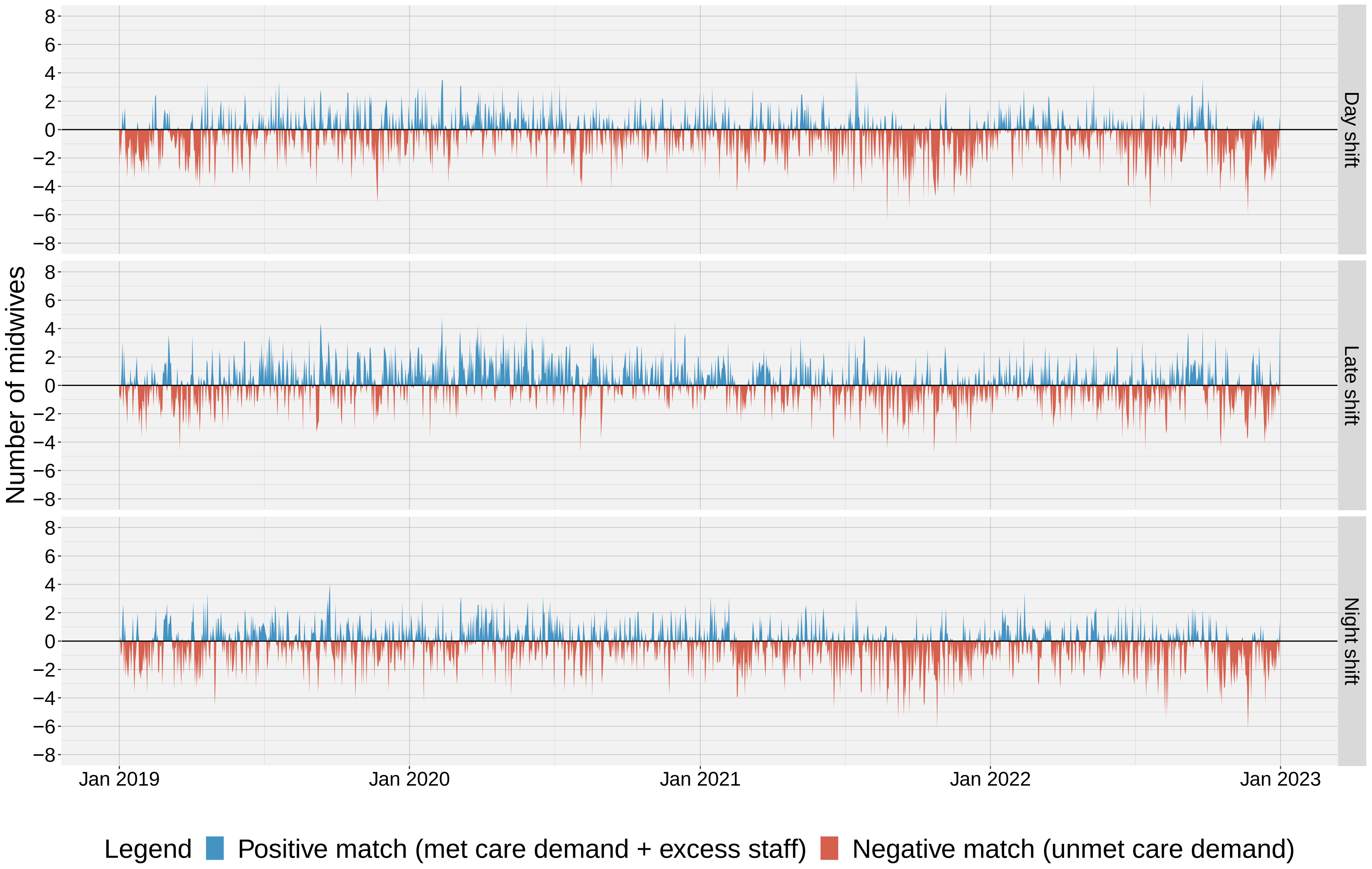

Figure S8 Match of staffing resources with unweighted care demand in Unit 2 (labour ward). Target ratio for all shifts 1:1, care demand is met at horizontal line = 0, number of registered midwives per shift who are either not needed (blue), or demand that is not met (red) in Unit 2, unweighted.

Figure S9

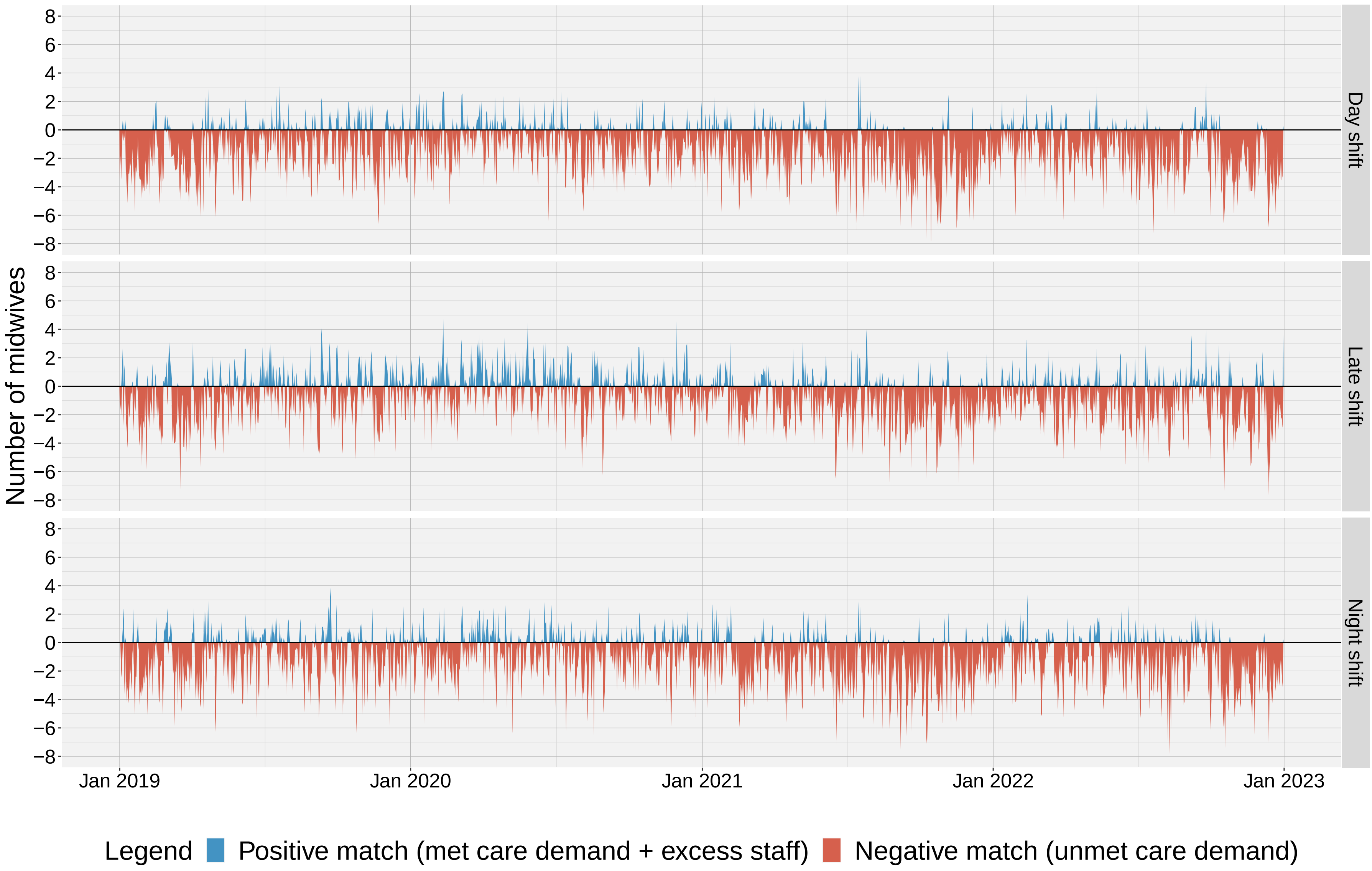

Figure S9 Match of staffing resources with weighted care demand in Unit 2 (labour ward). Target ratio for all shifts 1:1, care demand is met at horizontal line = 0, number of registered midwives per shift who are either not needed (blue), or demand that is not met (red) in Unit 2, weighted with complexity factor.

Figure S10

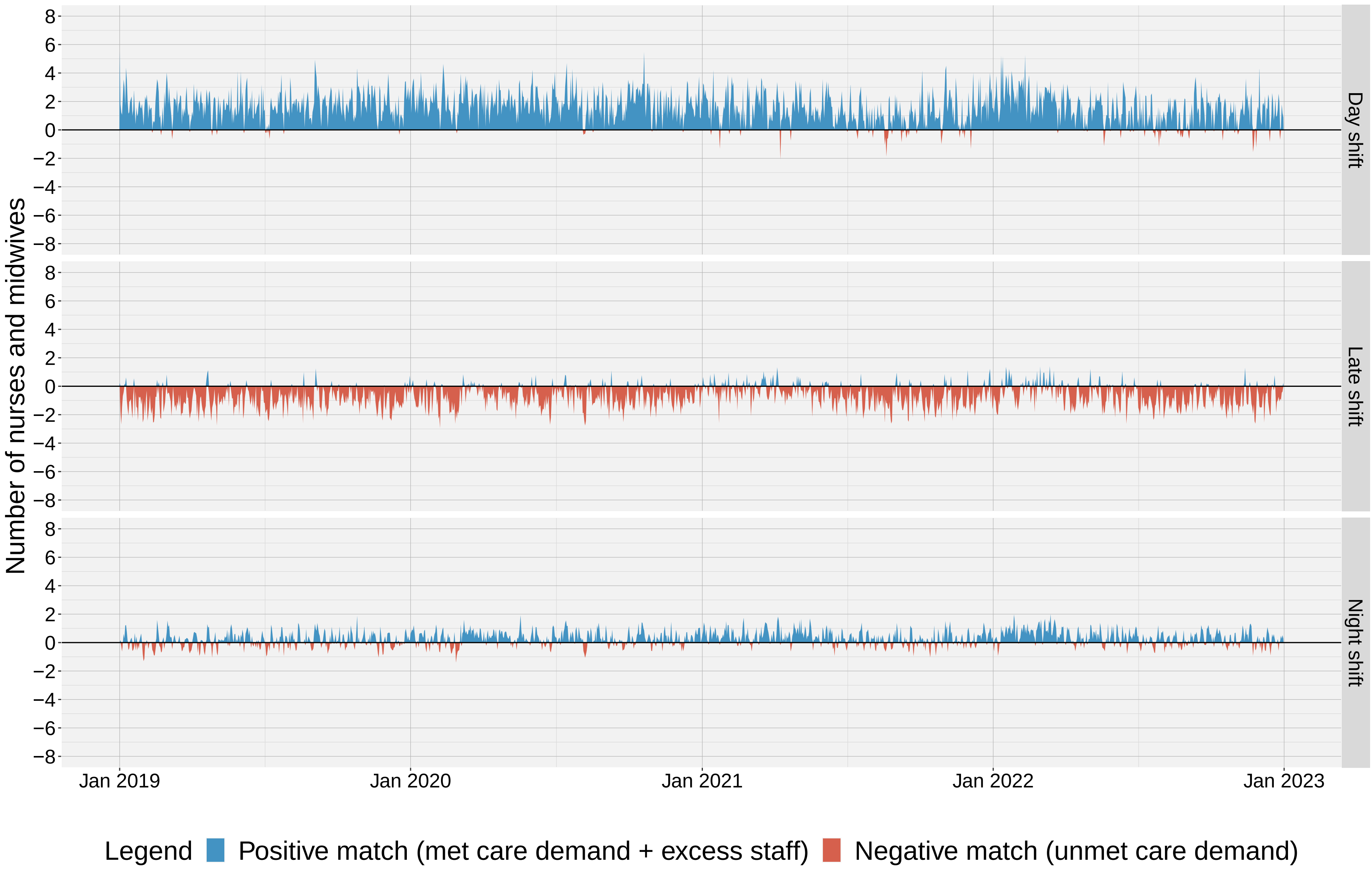

Figure S10 Match of staffing resources with unweighted care demand in Unit 3 (postnatal unit). Target ratios day shift 1:4, late shift 1:4, night shift 1:6, care demand is met at horizontal line = 0, number of registered midwives / nurses per shift who are either not needed (blue), or demand that is not met (red) in Unit 3, unweighted.

Figure S11

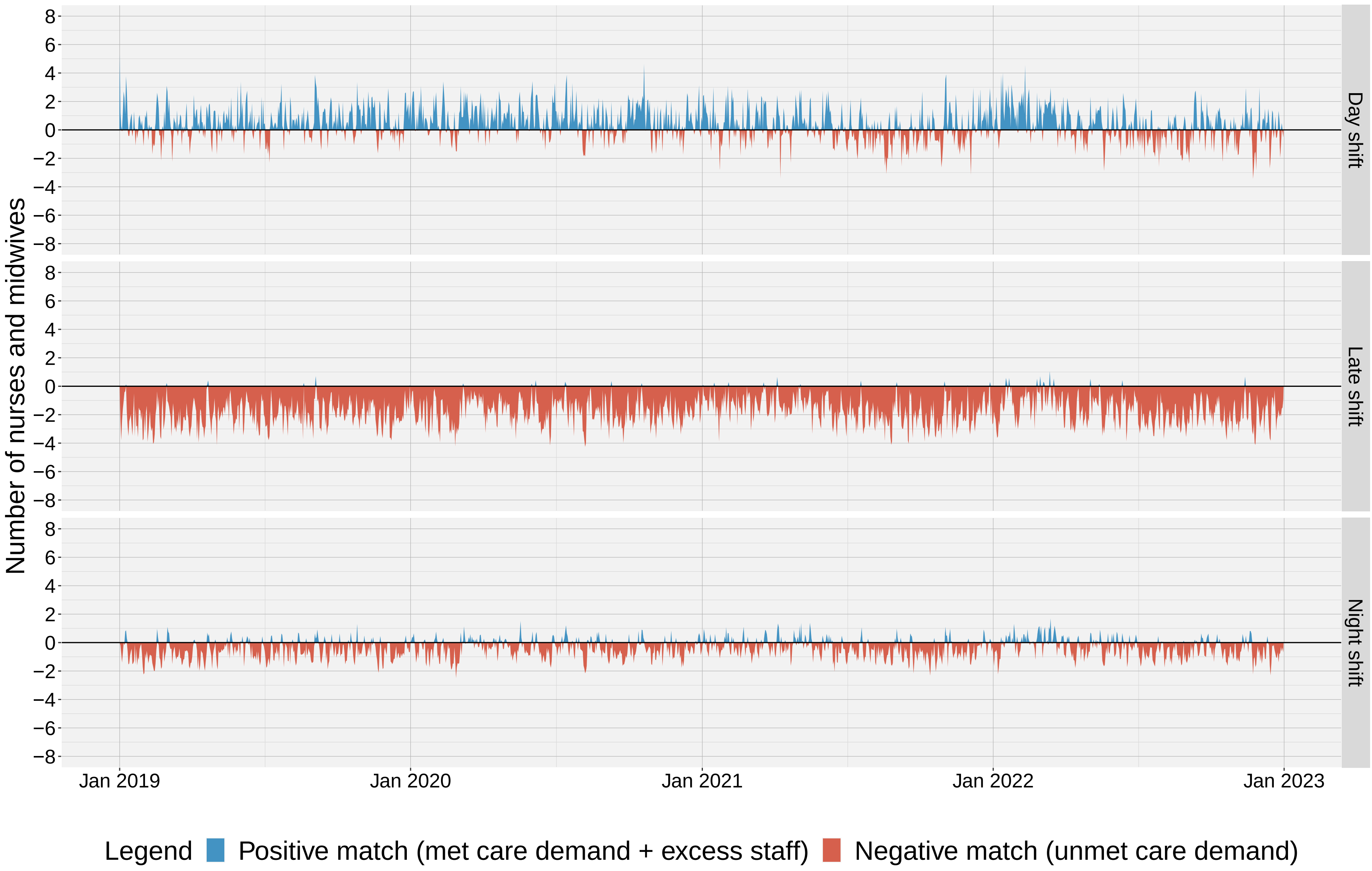

Figure S11 Match of staffing resources with weighted care demand in Unit 3 (postnatal unit). Target ratios day shift 1:4, late shift 1:4, night shift 1:6, care demand is met at horizontal line = 0, number of registered midwives / nurses per shift who are either not needed (blue), or demand that is not met (red) in Unit 3, weighted with complexity factor.

Figure S12

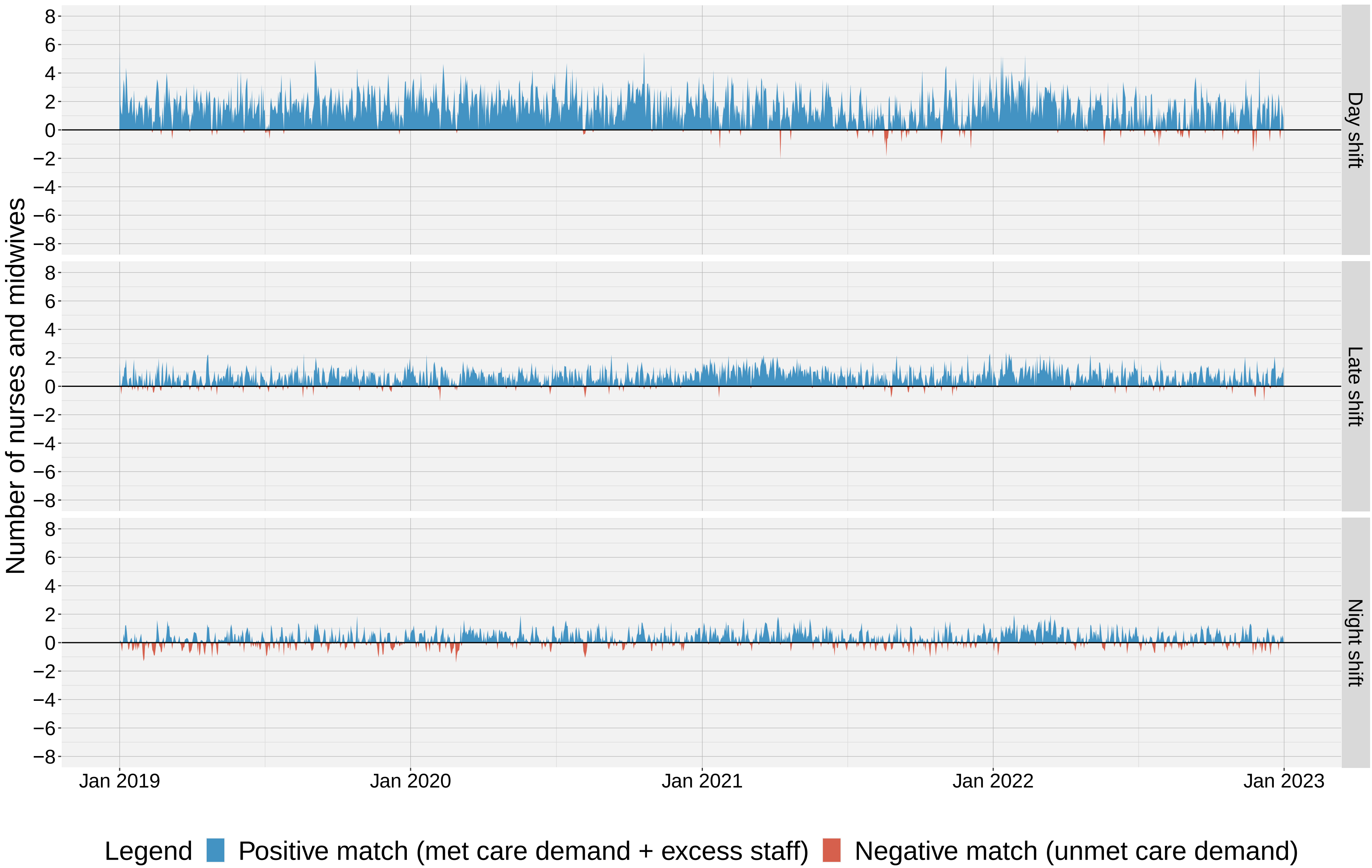

Figure S12 Match of staffing resources with unweighted care demand in Unit 3 (postnatal unit, unit-specific ratios). Target ratios day shift 1:4, late shift 1:6, night shift 1:6, care demand is met at horizontal line = 0, number of registered midwives / nurses per shift who are either not needed (blue), or demand that is not met (red) in Unit 3, unweighted.

Figure S13

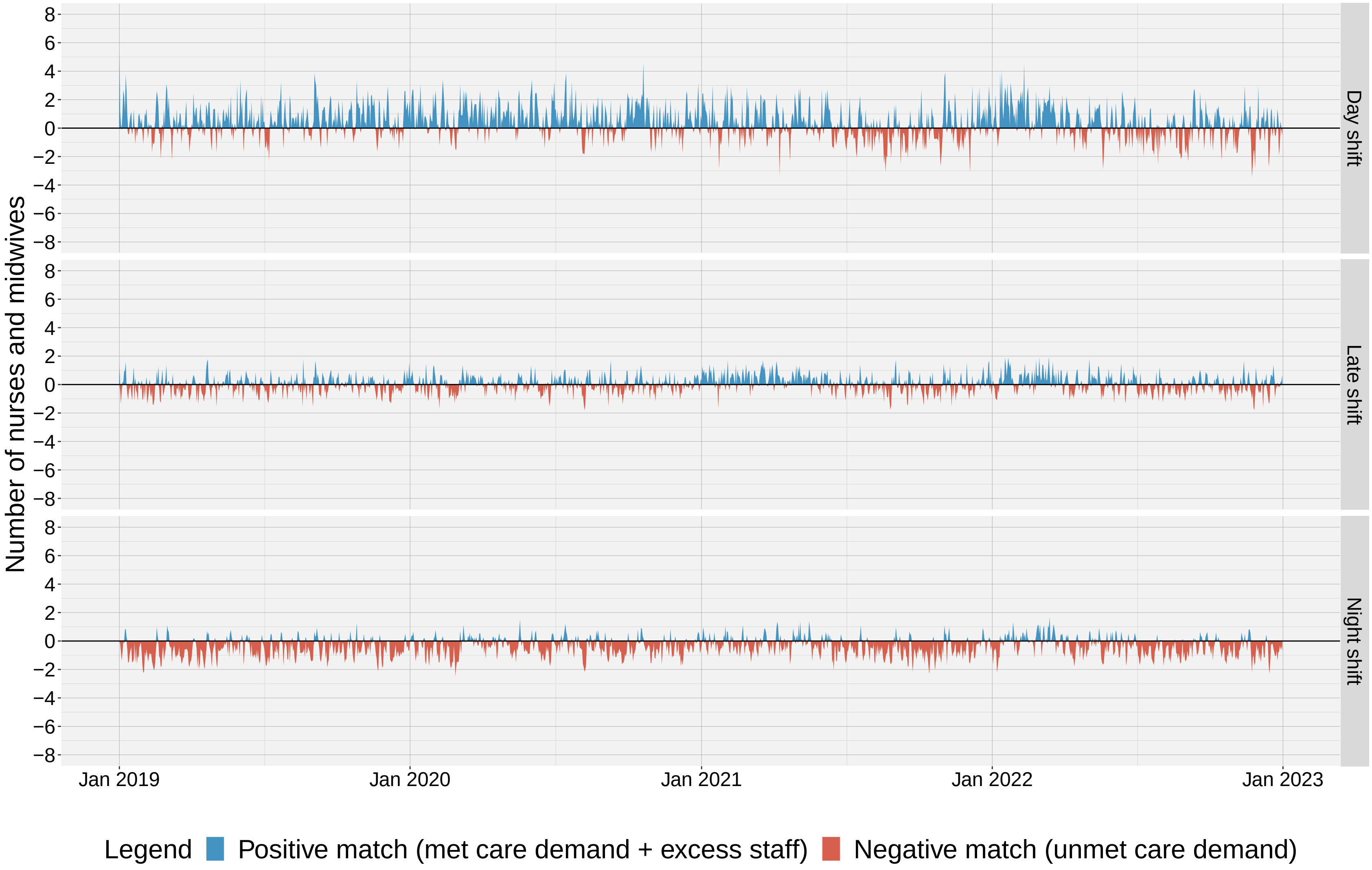

Figure S13 Match of staffing resources with weighted care demand in Unit 3 (postnatal unit, unit-specific ratios). Target ratios day shift 1:4, late shift 1:6, night shift 1:6, care demand is met at horizontal line = 0, number of registered midwives / nurses per shift who are either not needed (blue), or demand that is not met (red) in Unit 3, weighted with complexity factor.
